# The m^6^A eraser FTO facilitates proliferation and migration of human cervical cancer cells

**DOI:** 10.1186/s12935-019-1045-1

**Published:** 2019-12-02

**Authors:** Dongling Zou, Lei Dong, Chenying Li, Zhe Yin, Shuan Rao, Qi Zhou

**Affiliations:** 1 Chongqing Key Laboratory of Translational Research for Cancer Metastasis and Individualized Treatment, Chongqing University Cancer Hospital & Chongqing Cancer Institute & Chongqing Cancer Hospital, Chongqing, 400030 China; 2 Department of Systems Biology, Beckman Research Institute of City of Hope, Monrovia, CA USA; 3 Department of Hematology, School of Medicine, The First Affiliated Hospital of Zhejiang University, Hangzhou, 310003 China; 4 Department of Thoracic Surgery, Nanfang Hospital, Southern Medical University, Guangzhou, 510515 China

**Keywords:** FTO, m^6^A, Demethylase, E2F1, Myc, Translation

## Abstract

**Background:**

Since FTO was recognized as the first m^6^A demethylase, the understanding of its biological function has been widely expanded. However, the role of FTO in cervical cancer tumorigenesis remains unclear.

**Methods:**

In this study, we first analyzed the expression of FTO in two independent human cancer datasets and evaluated the correlation between FTO level and cervical cancer progression. Using small hairpin RNA technology, we explored the function of FTO in cervical cancer cell line Hela and SiHa cells, respectively. We then determined the FTO targets by performing transcriptional profile with FTO deficient and competent Hela cells, and finally validated these targets with ribosome profiling and functional rescue experiments.

**Results:**

Our data suggested that FTO was frequently overexpressed in human cervical cancer tissues and highly correlated with cervical cancer progression. FTO serves as an oncogenic regulator for cervical cancer cells’ proliferation and migration which is vastly depended on its demethylase activity. Mechanistically, FTO interacts with transcripts of E2F1 and Myc, inhibition of FTO significantly impairs the translation efficiency of E2F1 and Myc, however, either overexpress E2F1 or Myc sufficiently compensates the FTO deficiency which decreases cell proliferation and migration.

**Conclusions:**

Our study indicates that FTO plays important oncogenic role in regulating cervical cancer cells’ proliferation and migration via controlling m^6^A modification of E2F1 and Myc transcripts. FTO represents a potential drug candidate for cervical cancer therapy.

## Background

Cervical cancer is one of the most prevalent gynecological cancer worldwide, ranking 4th among all female cancers and affects about 450,000 newly diagnosed cases every year [1, 2]. Despite the advances in the screening and early prevention of cervical cancer dramatically decreases the overall cervical cancer incidence and mortality in United States and other western countries [3], a vast majority of patients in low and low-middle income countries are still diagnosed as locally advanced or invasive cervical cancer with high risks of morbidity, metastasis as well as recurrence [4]. Therefore, it is necessary to seek novel biomarkers for early detection and new targets for improved cervical cancer’s therapy and prognosis.

The *N*^6^-methyladenosine (m^6^A) is the most abundant eukaryotic messenger RNA (mRNA) modification which was discovered in the 1970s [5, 6]. Recently, the fat mass- and obesity-associated protein (FTO) was identified as the first RNA demethylase [7], expanding the knowledge that the m^6^A is a reversible and dynamic process which can regulate not only mRNA stability, splicing, transport, localization and translation, but also microRNA processing and RNA-protein interaction [8–20]. Till now, the m^6^A networking is well characterized into three subtypes, including “writers”, “readers” and “erasers”. Briefly, METTL3 and METTL4 are “writers” that form a heterodimer to catalyze m^6^A methylation together with WTAP [16, 21–23]; YTHDF1/2/3 are identified as “readers” that bind to RNA sequences with m^6^A sites and controlled different biological functions, such as RNA decay or mRNA translation [10, 15, 17], the “erasers” include FTO [7] and another RNA demethylase ALKBH5 [19], which catalyze m^6^A demethylation in a Fe(II)- and a-ketoglutarate-dependent manner. The dysfunction of these m^6^A regulators has been implicated to be involved in different physiological and pathological process, particularly in cancer initiation and progression [24, 25].

FTO was originally identified as a regulator for body mass and obesity, as FTO’s deficiency resulted in growth retardation while overactivation of FTO increased food intake and led to obesity [26–28]. After recognized as the first RNA demethylase, novel functions of FTO have been discovered to control different aspects of biological process, such as dopaminergic signaling and adipogenesis [18, 29, 30]. More importantly, increasing proofs reveal that dysfunction of FTO can contribute to cancer development, such as acute myeloid leukemia [31], melanoma [32], breast cancer [33] and lung cancer [34].

In this study, we aim to explore the expression of FTO and its correlation with cervical cancer progression, moreover, we sought to uncover the detail molecular mechanisms on how FTO regulate cervical cancer progression.

## Methods

### Clinical specimens and cell lines

Primary cervical cancer tissues and normal cervical tissues were collected from Chongqing University Cancer Hospital from 2010 to 2019. Two cervical cancer cell line, Hela and SiHa were obtained from the ATCC and grown in DMEM with 10% FBS (Hyclone, Logan, Utah) at 37 °C in 5% CO_2_ cell culture incubator. Cell lines were tested for mycoplasma detection according to the ATCC cell line verification test recommendations.

### Plasmid construction and transfections

The pGFP-C-shFTO and pGFP-C-shNC were purchased from Origene technologies (Rockville, MD). The pcDNA3.1_FTO and pcDNA3.1_mutFTO constructs were kindly provided by Dr. Jianjun Chen. The E2F-CDS and Myc-CDS were amplified from human genomic cDNA and directly inserted into pcDNA3.1 vector. The PCR primers were described in Table 1. The above plasmids were transfected into Hela and SiHa cells using Lipofectamine LTX (Thermo Fisher) according to the protocol. The efficiency of transfection was confirmed by western blot.

**Table 1:** Primers used in qRT-PCR or constructs

Primer	Sequence(5′–3′)
FTO-forward	CCCTGTGAGCAGCAACATAAG
FTO-reverse	CAACCCGACCCAGTCTAAATC
E2F-forward	GCTCTGTTCCCTCCTGCTTT
E2F-reverse	CAAATCAAATCGGGCACG
E2F-CDS F	ATGGCCTTGGCCGGGGCCCC
E2F-CDS R	TCAGAAATCCAGGGGGGTGAGG
Myc-forward	GCCTTGGTTCATCTGGGTCTA
Myc-reverse	TGCTTAGGAGTGCTTGGGAC
Myc-CDS F	CTGGATTTTTTTCGGGTAGT
Myc-CDS R	TTACGCACAAGAGTTCCGTAGC

### RNA extraction and quantitative RT-PCR analysis

Total RNAs were isolated using the miRNeasy kit (Qiagen, Valencia, CA). For mRNA expression, 200 ng RNA was reverse transcribed into cDNA in a total reaction volume of 10 μL with Qiagen’s RT kit according to the manufacturer’s instructions. Quantitative real-time PCR analysis was performed with 0.5 μL cDNA using SYBR green PCR master mix (Qiagen, Valencia, CA) in an AB7900HT real-time PCR instrument (Applied Biosystems, Foster City, CA). Gapdh or Actin was used as endogenous loading control. Each sample was run in triplicates. Primer sequences are listed in Table 1.

### Immunoblotting (Western blot)

Cells were washed twice with ice-cold phosphate-buffered saline (PBS) and ruptured with RIPA buffer (Pierce, Rockford, IL) containing 5 mM EDTA, PMSF, cocktail inhibitor, and phosphatase inhibitor cocktail. Cell extracts were micro centrifuged for 20 min at 10,000×*g* and supernatants were collected. Cell lysates (20 μL) were resolved by SDS-PAGE and transferred onto PVDF membranes. Membranes were blocked for 1 h with 5% skim milk in Tris buffered saline containing 0.1% Tween 20 and incubated overnight at 4 °C with anti-FTO antibody (ab124892, Abcam), anti-E2F antibody (ab179445, Abcam), anti-Myc (ab32072, Abcam) and Anti-GAPDH (60004-1-Ig, Proteintech). Membranes were washed 30 min with Tris-buffered saline containing 0.1% Tween 20, incubated for 1 h with appropriate secondary antibodies conjugated to horseradish peroxidase, and developed using chemiluminescent substrates.

### m^6^A dot blot assay

Total RNA was isolated from different cells with miRNeasy Mini Kit (QIAGEN, 217004) according to the manufacturer’s instructions and quantified by UV spectrophotometry. The m^6^A dot blot assay was performed following a published protocol with some modifications [7]. Briefly, the RNA samples were loaded to the Amersham Hybond-N+ membrane (RPN119B, GE Healthcare) with a Bio-Dot Apparatus (#170-6545, Bio-Rad) and UV crosslinked to the membrane. Then the membrane was blocked with 5% nonfat dry milk (in 1× PBST) for 1–2 h and incubated with a specific anti-m^6^A antibody (1:2000 dilution, Synaptic Systems, 202003) overnight at 4 °C. Then the HRP-conjugated goat anti-rabbit IgG (sc-2030, Santa Cruz Biotechnology) was added to the blots for 1 h at room temperature and the membrane was developed with Amersham ECL Prime Western Blotting Detection Reagent (RPN2232, GE Healthcare). The relative signal density of each dot was quantified by Gel-Pro analyzer software (Media Cybernetics) in all experiments.

### Cell proliferation and invasion assays

To measure the cellular proliferation rates, Hela or SiHa cells were incubated in 10% CCK-8 (DOJINDO) diluted in normal culture media at 37 °C until visual color conversion appears. Proliferation rates were determined at 12, 24, 36, 48, 60, 72 h post-transfection and quantification was done on a microtiter plate reader (Spectra Rainbow, Tecan) according to the manufacturer-recommended protocol, respectively. For cell invasion assay, cervical cancer cells were seeded onto a Matrigel-coated membrane matrix (BD Bioscience) present in the insert of a 24 well culture plate. Fetal bovine serum was added to the lower chamber as a chemoattractant. After 24 h, the non-invading cells were gently removed with a cotton swab. Invasive cells located on the lower surface of chamber were stained with the 0.1% crystal violet (Sigma) and counted.

### RNA immunoprecipitation

To enable RNA immunoprecipitation, 50 μL of protein A Sepharose beads were incubated with 5 μg of anti-FTO antibody (ab124892, Abcam) or m^6^A antibody (Synaptic Systems, 202003) or pre-immune IgG in 500 μL wash buffer (20 mM HEPES PH 7.9, 150 mM NaCl, 0.5 mM EDTA PH 8.0, 10 mM KCl, 1.5 M MgCl_2_, 0.5% Np-40, 10% glycerine, 1.5 mM DTT, 1 mM PMSF) at 4 °C for 4–6 h. Then the beads were washed three times with wash buffer and kept on ice until use. Harvested Hela and SiHa cells were suspended in 400 μL lysis buffer (20 mM HEPES PH 7.9, 150 mM NaCl, 0.5 mM EDTA PH 8.0, 10 mM KCl, 1.5 M MgCl_2_, 0.5% Np-40, 10% glycerine, 1.5 mM DTT, 1× Protease Inhibitor cocktail (Roche), 10 U mL^−1^ RNase Inhibitor) and then left on ice for 30 min followed by centrifugation. The supernatant was transferred to tubes with antibody or pre-immune IgG-coated beads, and IP was performed by tube rotating at 4 °C overnight. Following IP, the beads were washed two times. Finally, the RNA was extracted with Trizol reagent (Invitrogen, CA, USA).

### Polysome profiling

Prior to harvesting, Hela and SiHa cells were treated with 100 μg mL^−1^ cycloheximide (CHX) for 10 min at 37 °C. Cells were washed in phosphate-buffered saline (PBS) and resuspended in hypotonic lysis buffer (20 mM Tris pH 7.5, 5 mM MgCl_2_, 100 mM KCl, 0.5% NP-40, 0.5 mM β-mercaptoethanol, 40 U mL^−1^ RNase inhibitor, protease inhibitor cocktail, 1 mM PMSF, 100 μg mL^−1^ CHX and incubated on ice for 15 min. The cytoplasmic fraction was extracted by low-speed centrifugation and loaded onto a 10–50% sucrose gradient. Gradients were centrifuged in an SW40 rotor at 35,000 rpm for 2 h. Gradients were fractionated using an Isco Fractionator by piercing the bottom of the tube and chasing the gradient with a 60% sucrose solution (60% sucrose (w/v), 20 mM Tris pH 7.5, 5 mM MgCl_2_, 1 mM KCl, 40 U mL^−1^ RNase inhibitor, protease inhibitor cocktail, 1 mM PMSF. Fractions were collected with concomitant measurement of the OD 254 nm. RNA was isolated and analyzed as previously mentioned.

### Analysis of RNA sequencing data

Hela cells were transfected with pGFP-C-shFTO or pGFP-C-shNC for 48 h and total RNA was prepared for next-generation sequencing (NGS). The NGS library preparation was constructed by TruSeq Stranded mRNA Sample Prep Kit (Illumina) and was quantified by Bio Analyzer High Sensitivity DNA chip, and then was deeply sequenced on the Illumina HiSeq 2500. The data have been deposited in the GEO repository with the accession numbers GSE133517. The expression level of genes was quantified using RSEM in TPM format. The differentially expressed genes with FTO knockdown were shown in Additional file 1: Table S1.

### Statistical analysis

Statistical differences in tissue FTO mRNA expression levels between cancer and normal sample sources were determined using two-sided Mann–Whitney U test. Student’s t test (two-tailed) was performed for two-group data and three-group data were analyzed using one-way Anova analysis of variance. All data were analyzed using GraphPad Prism 5.0 software (GraphPad Software, Inc., USA) and presented as mean ± SD.

### Data availability

The RNA sequencing data were available from the GEO database under accession number GSE133517.

## Results

### FTO is overexpressed in cervical cancer and significantly correlated with cancer progression

**Fig. 1:**
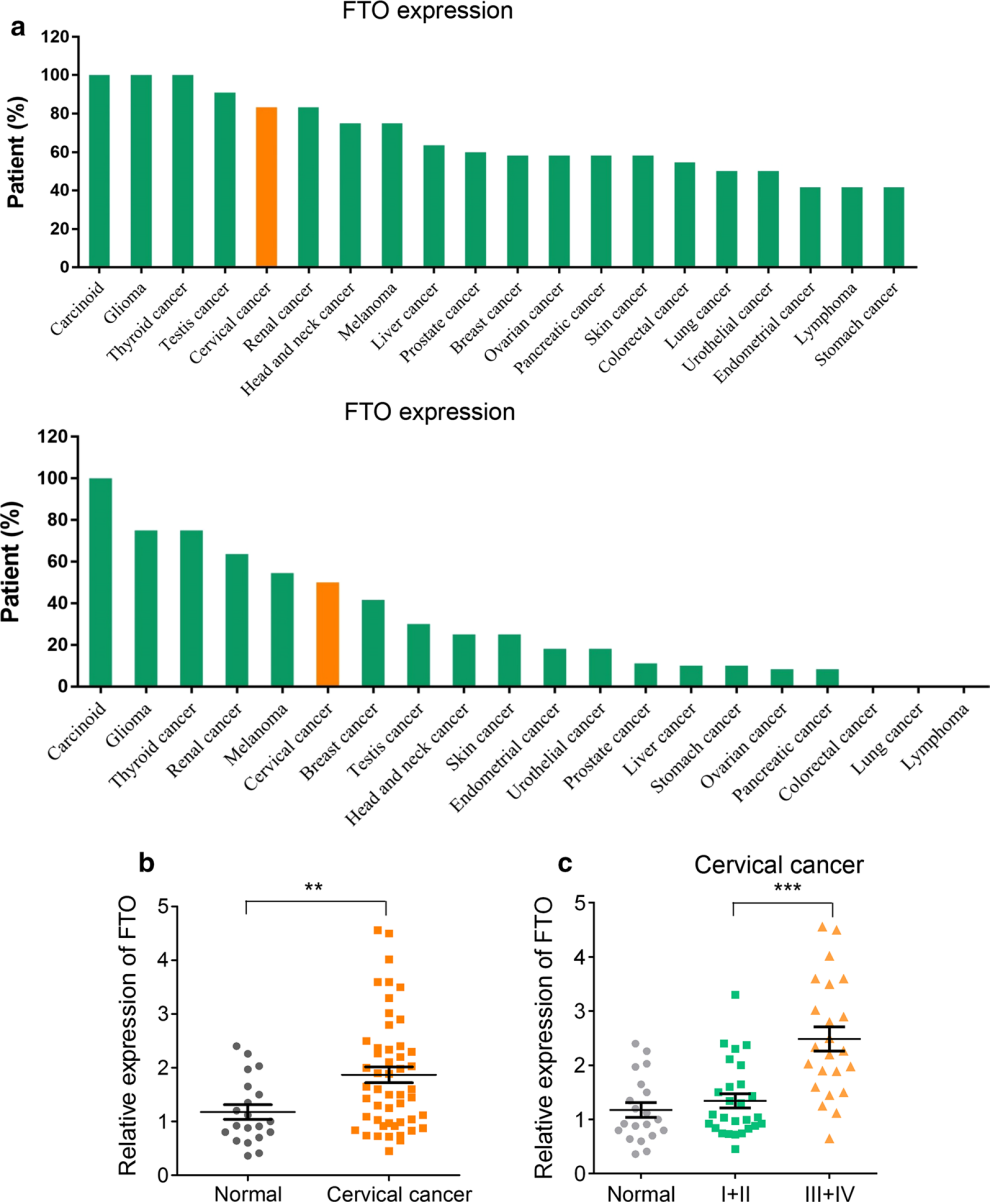
FTO is highly expressed in human cervical cancer. **a** Evaluation of relative FTO expression in different cancer subtypes using HPA041086 (upper panel) and HPA068695 (lower panel). **b** qRT-PCR analysis of FTO expression in 20 cervical normal cervix tissues and 50 cervical cancer patients’ tissues. **P < 0.01 (two-sided Mann–Whitney U test). **c** The FTO expression analysis in normal cervices tissues, early (phase I and II, n = 28) and late (phase III and IV, n = 22) stage cervical cancer patients.***P < 0.001 (two-sided Mann–Whitney U test)

To get an overview of FTO’s expression pattern in human cervical cancer tissues, we first examined FTO’s protein levels in different human cancer tissues by analyzing two independent human datasets HPA041086 (Fig. 1a, upper panel) and HPA068695 (Fig. 1a, lower panel). Indeed, FTO’s high expression was more frequently identified in cervical cancer tissues compared to many other cancer subtypes. Next, using in-house collected cervical cancer samples, we investigated the relative transcription difference of FTO between normal cervices and cervical cancer tissues, indeed, our data revealed that FTO was significantly higher expressed in cervical cancer tissues than normal controls (Fig. 1b). Moreover, when distributed all cervical cancer patients according to their clinical stage, we observed a dramatically higher FTO’s expression in late stage patients (stage III and IV) compared to early stage patients (stage I and II) as well as normal cervices, suggesting upregulation of FTO was usually correlated with cervical cancer progression (Fig. 1c).

### FTO facilitates cervical cancer cells’ proliferation and migration

**Fig. 2:**
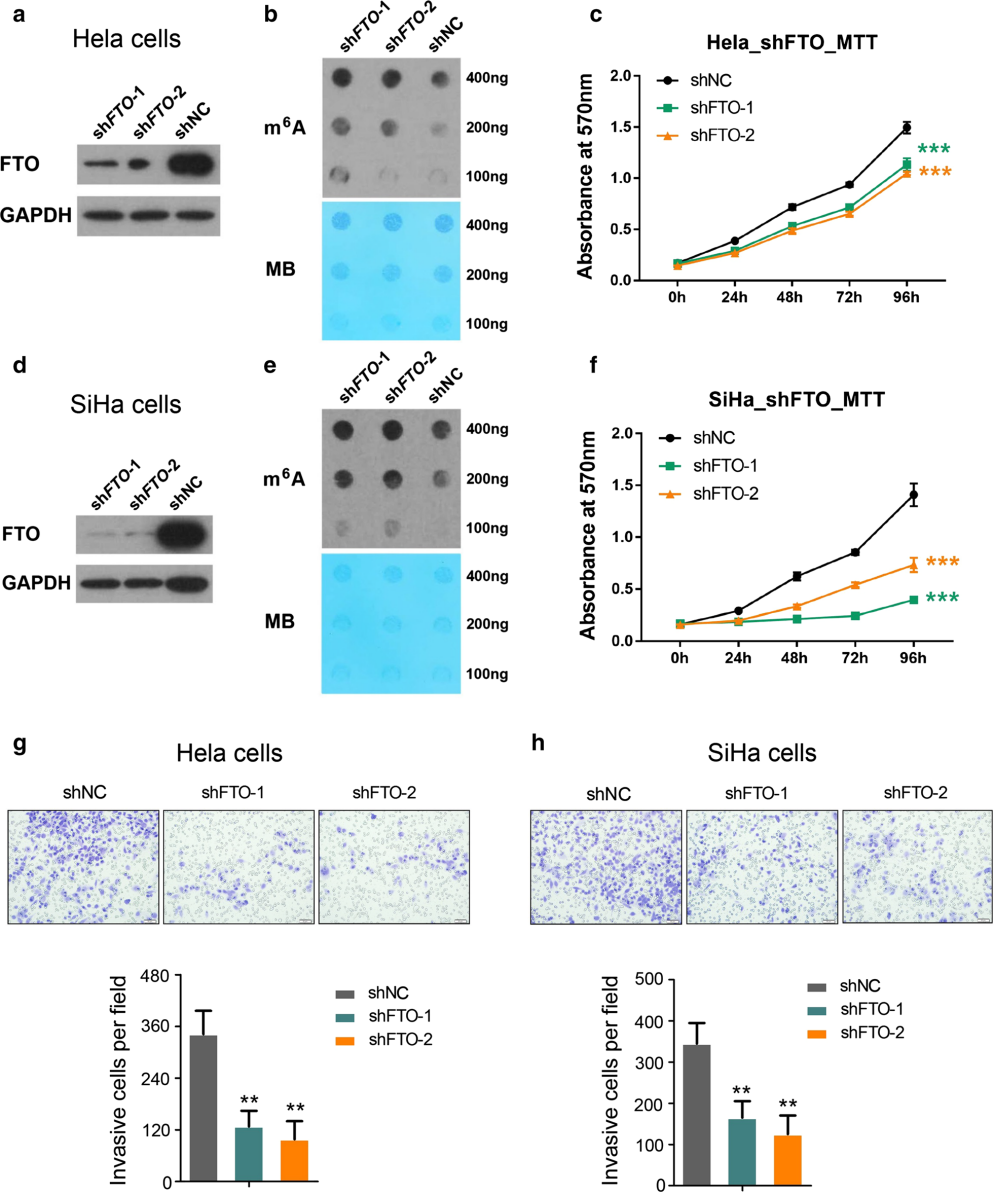
FTO regulates cervical cancer cells’ proliferation and migration. **a** Immunoblot analysis of FTO expression in control and knocking down Hela cells using two different shRNAs; **b** m^6^A dot blot assays of Hela cells with or without knocking down of FTO. MB, methylene blue staining (as loading control); **c** effects of knocking down FTO on Hela cells growth/proliferation. ***P < 0.001. (Student’s t test); **d** Western blot analysis of FTO expression in control and knocking down SiHa cells using same shRNAs as described in **a**; **e** m^6^A dot blot assays of SiHa cells with or without knocking down of FTO. MB, methylene blue staining (as loading control); **f** effects of knocking down FTO on SiHa cells growth/proliferation. ***P < 0.001. (Student’s t test); **g**, **h** analysis of cell migration capacity using competent or deficient Hela and SiHa cells

As FTO is frequently overexpressed in human cervical cancer tissues, we then explored the detail function of FTO in tumorigenesis with loss-of-function study by introducing two FTO specific small hairpin RNAs (shRNAs) into Hela cells, respectively. The FTO’s expression was efficiently suppressed (Fig. 2a), resulting in obvious upregulation of global mRNA m^6^A level (Fig. 2b) and significant inhibition of cell proliferation (Fig. 2c); the similar effect was reproduced with another human cervical cancer cell line, SiHa cells (Fig. 2d–f). Furthermore, we also observed the migration capacity of Hela and SiHa cells was dramatically impaired when FTO was deficient (Fig. 2g, h), indicating FTO was an important factor which positively regulated cervical cancer cells’ proliferation and migration.

### The oncogenic function of FTO is m^6^A RNA demethylase dependent

**Fig. 3:**
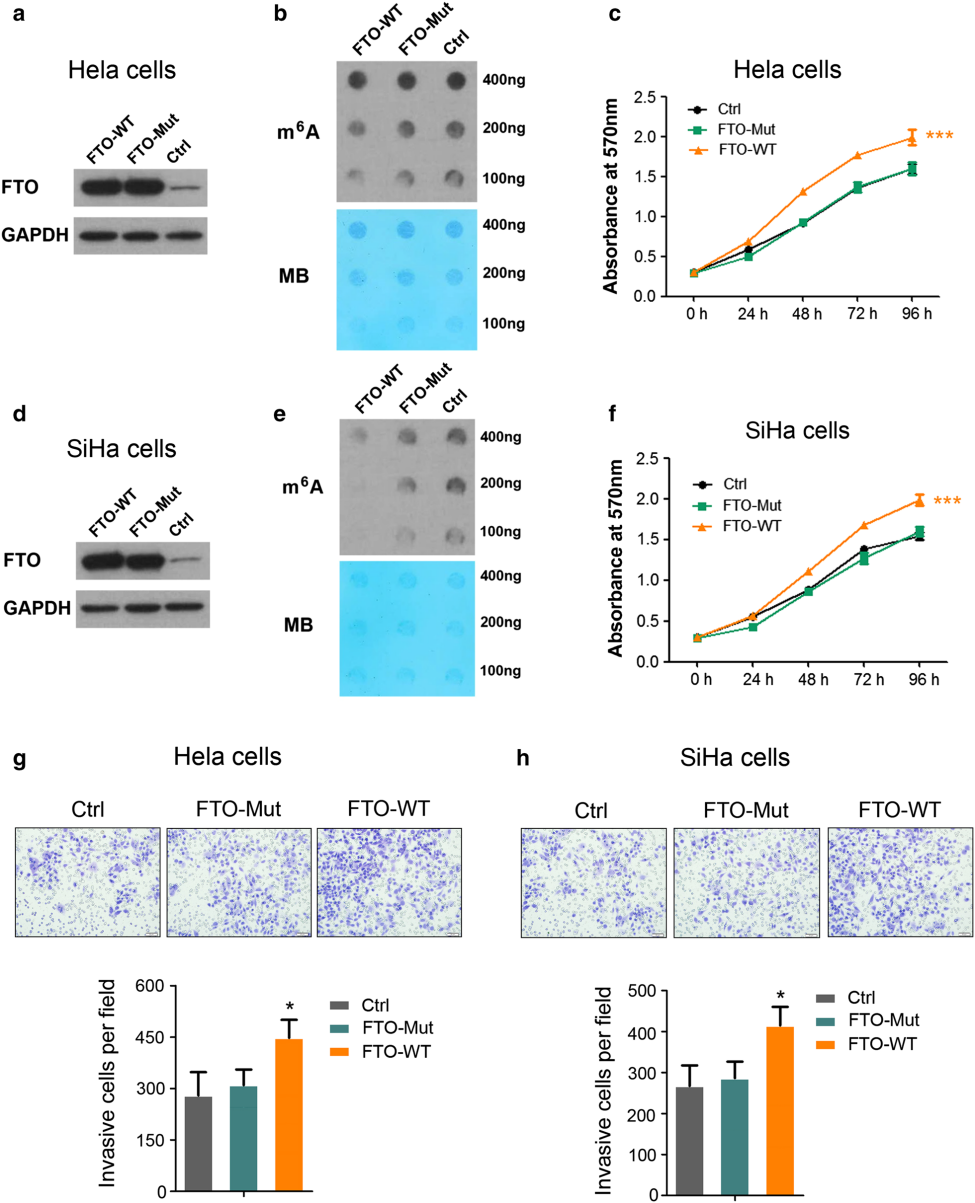
The m^6^A demethylase activity is required for FTO to play its oncogenic function. **a** Enforced FTO or FTO-mut expression in Hela cells. FTO-mut carries two point- mutations, H231A and D233A, which disrupt the enzymatic activity of FTO. GAPDH was used as a loading control; **b** m^6^A dot blot assays of Hela cells with enforced FTO or FTO-mut expression. MB, methylene blue staining (as loading control); **c** effects of FTO or mutant FTO overexpression on Hela cells’ growth/proliferation, ***P < 0.001. (Student’s t test); **d** enforced FTO or FTO-mut expression in SiHa cells. GAPDH was used as a loading control; **e** m^6^A dot blot assays of SiHa cells with enforced FTO or FTO-mut expression. MB, methylene blue staining (as loading control); **f** effects of FTO or mutant FTO overexpression on SiHa cells’ growth/proliferation, ***P < 0.001. (Student’s t test); **g**, **h** analysis of cell migration capacity in FTO or mutant FTO overexpressed Hela and SiHa cells

We next questioned whether FTO’s RNA demethylase activity was essential to play its oncogenic role in cervical cancer tumorigenesis. Forced expression of wildtype FTO (FTO-WT) and mutant FTO (FTO-Mut, with H231A and D233A two point-mutations which abolished FTO enzymatic activity) [31] was achieved by lentiviral transduction into Hela cells (Fig. 3a). The total mRNA m^6^A level was moderate decreased due to the endogenous expression of FTO (Fig. 3b) in both FTO-Mut and control cells, however, cell proliferation was significantly increased in FTO-WT but not FTO-Mut cells (Fig. 3c), suggesting the enzymatic activity of FTO as a RNA demethylase was detrimental to regulate cervical cells proliferation. Again, we observed comparable phenomenon with SiHa cells using same strategy (Fig. 3d–f). The migration assay also revealed that demethylation activity of FTO was required to regulate cell movement as overexpression of FTO-WT but not FTO-Mut could efficiently increase cervical cancer cells’ migration (Fig. 3g, h).

### Identification of FTO targets

**Fig. 4:**
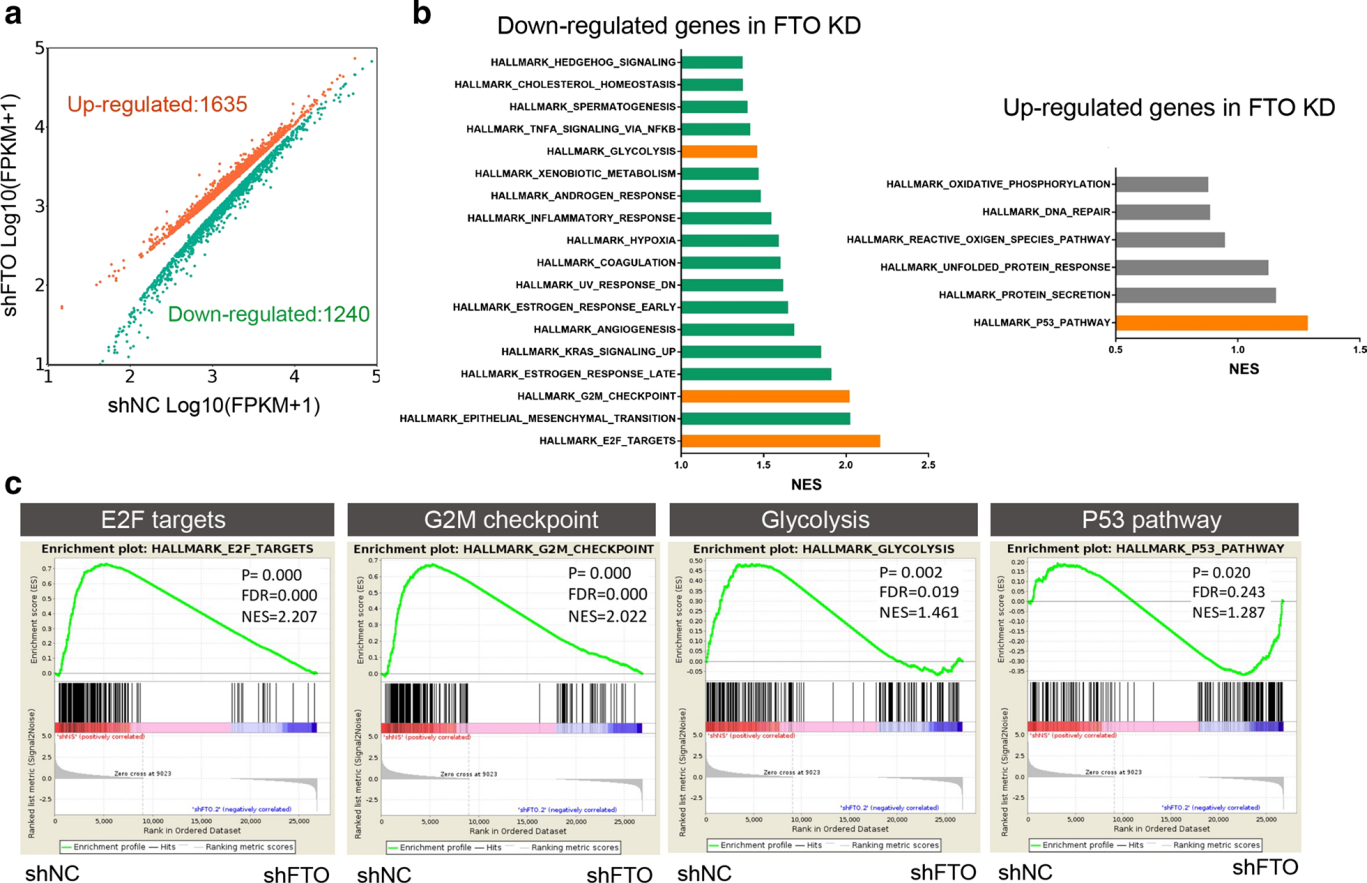
Identification of FTO targeted genes. **a** Volcano plots showed 1635 up-regulated genes and 1240 down-regulated genes in FTO deficient Hela cells; **b** gene ontology analysis revealed up-regulated and down-regulated pathways in FTO knocking down cells; **c** gene set enrichment analysis (GSEA) of Hela cells with or without FTO deficiency

To comprehensively understand the global gene expression changes after suppressing FTO, we performed RNA-seq profiling analysis with control and FTO knockdown Hela cells, 1635 upregulated genes as well as 1240 downregulated genes was identified (Fig. 4a). Gene ontology (GO) analysis suggested that most downregulated genes upon knocking down FTO were enriched in cancer associated pathways, such as E2F1 targets, Epithelial mesenchymal transition (EMT), cell cycle regulation and glycolysis; in contrast, most upregulated genes were enriched in tumor suppressing pathways, including p53 pathway, unfolded protein response (UPR) and DNA damage repairs (Fig. 4b), these findings further supported the notion that FTO was a oncogenic factor contributed to cervical cancer progression. Next, we performed gene set enrichment analysis (GSEA) and observed E2F1 targets, G2M checkpoint regulators, glycolysis was downregulated while p53 pathway was upregulated in FTO knocking down cells (Fig. 4c). Since E2F1 and Myc are key players for E2F1 targets and cell cycle regulation, and these two genes were repeatedly identified by different enrichment assays, we therefore chose these two proteins as the potential targets of FTO for further analysis.

### The translation of E2F1 and Myc is regulated by FTO

**Fig. 5:**
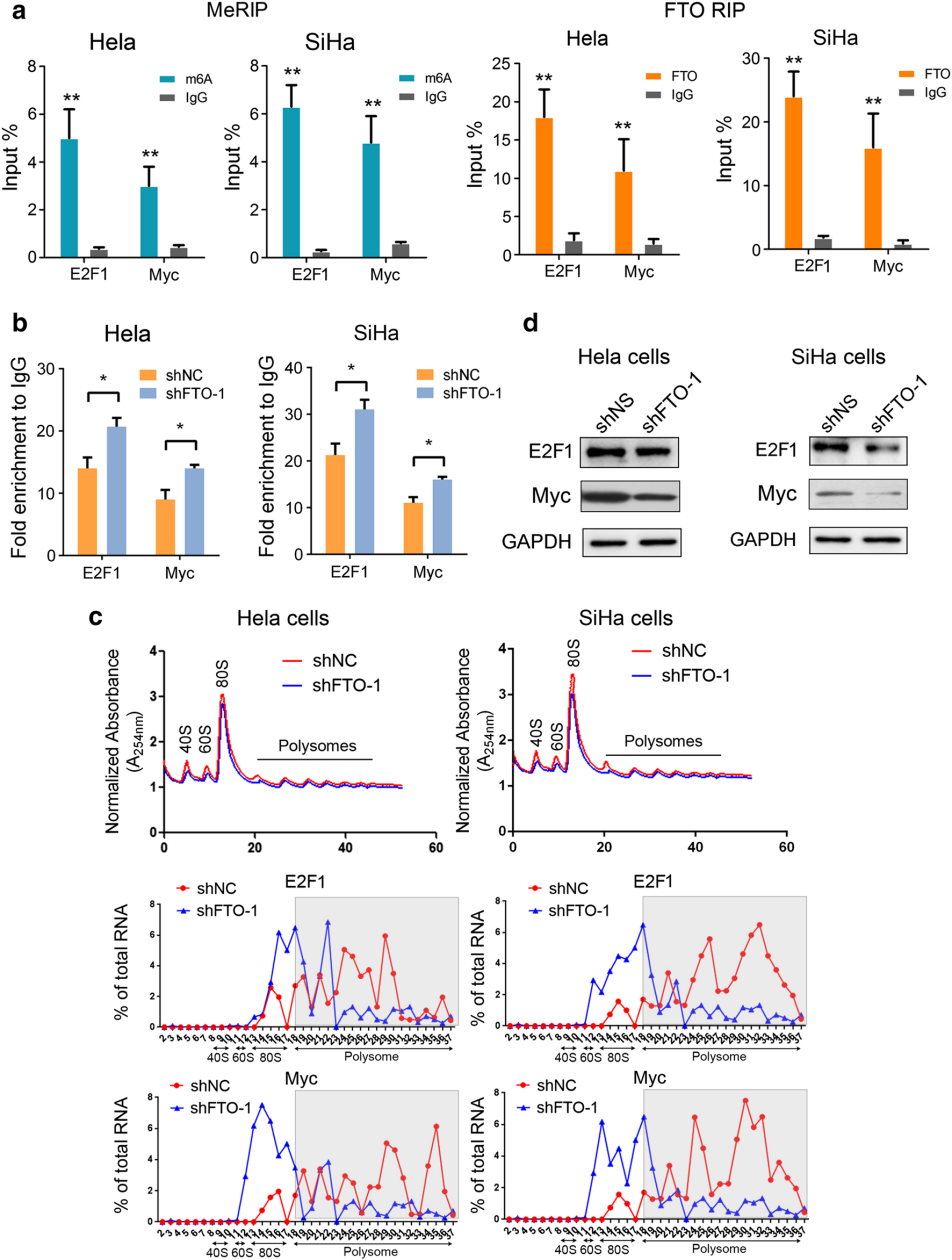
E2F1 and Myc are direct downstream targets of FTO. **a** Methylated RNA immunoprecipitation of the transcripts of E2F1 and Myc (left panel) and FTO immunoprecipitation assay of E2F1 and Myc transcripts in FTO bound mRNAs (right panel), IgG was used as an internal control. **b** Gene-specific m^6^A qPCR analysis of m^6^A level of E2F1 and Myc transcripts in FTO control and knockdown Hela and SiHa cells. **c** Polysome profiling of FTO competent and deficient Hela and SiHa cells (upper panel); qRT-PCR examination of E2F1 (middle panel) and Myc (lower panel) mRNA distribution in different ribosome populations. **d** Western blot analysis of E2F1 and Myc expression in FTO knocking down Hela and SiHa cells

To investigate whether direct interaction was existed between FTO and E2F1 as well as Myc, we first performed methylated RNA immunoprecipitation (MeRIP) analysis in Hela and SiHa cells, which indicated that both transcripts of E2F1 and Myc were highly m^6^A modified (Fig. 5a); we then used an FTO specific antibody to conduct RIP analysis to identify FTO direct bound transcripts. Our data suggested that E2F1 and Myc’s mRNAs were significantly enriched in FTO regulated transcripts (Fig. 5a), all these results confirmed that the cervical cancer drivers such as E2F1 and Myc were dynamically modified by m^6^A methylation, while as an “eraser”, FTO could directly regulate these mRNAs demethylation as m^6^A modification in E2F1 and Myc transcripts were significantly increased when FTO was inhibited (Fig. 5b).

We next explored the general effect of knocking down FTO in Hela and SiHa cells, as revealed by polysome profiling, the global translation of FTO deficient cervical cancer cells was comparable to FTO competent controls (Fig. 5c), however, when we evaluated the translation of E2F1 and Myc specifically, we found the translation efficiency of E2F1 and Myc was dramatically impaired after inhibiting FTO (Fig. 5c), resulting decreased protein level of E2F1 and Myc in cervical cancer cells (Fig. 5d).

### Ecotopic expression of E2F1 or Myc compensates FTO’s deficiency

**Fig. 6:**
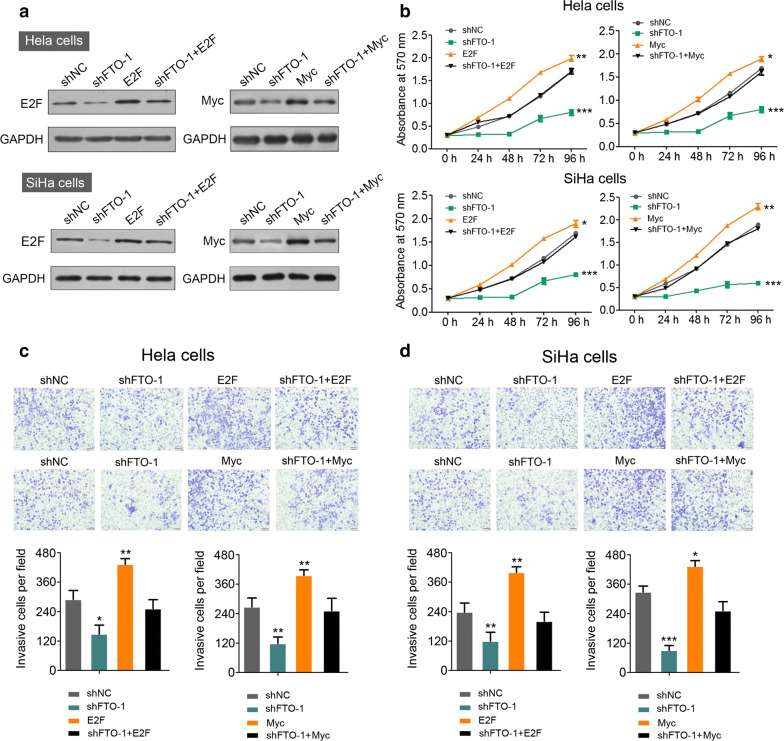
Overexpression of E2F1 or Myc compensates FTO knocking down effect. **a** The protein level determination of overexpressed E2F1 or Myc in FTO control and knockdown Hela and SiHa cells, **P < 0.01 (unpaired two-sided t test); **b** cell proliferation analysis of Hela (upper panel) and SiHa cells (lower panel) in different genetic background as described (**a**). *P < 0.05, **P < 0.01, ***P < 0.001 (Student’s t test). **c**, **d** The migration analysis of Hela cells (**c**) and SiHa cells (**d**) in different genetic background as described in **a**

So far, we demonstrated that FTO could directly regulate E2F1 and Myc’s translation, FTO deficiency impaired cells’ proliferation and migration. We then questioned whether introduction of ecotopic E2F1 or Myc’s expression could rescue the phenotype which induced by inhibiting FTO. E2F1 or Myc was successfully re-expressed in control and FTO knocking down Hela and SiHa cells (Fig. 6a). Interestingly, overexpress E2F1 or Myc could accelerate cervical cancer cells’ proliferation as compared to their normal controls, suggesting the oncogenic function of E2F1 and Myc in cervical cancer development (Fig. 6b); intriguingly, either overexpression of E2F1 or Myc in FTO deficient cervical cancer cells could completely re-establish the proliferation capacity to equivalent levels as compared to FTO functional control cells (Fig. 6b). Similarly, ecotopic expression of E2F1 or Myc could significantly increase FTO competent cervical cancer cells’ migration compare to control cells (Fig. 6c, d). Although inhibition of FTO suppressed cells’ movement, re-expression of either E2F1 or Myc could efficiently abolish such effect which induced by knocking down FTO, indicating FTO derived oncogenic effects are dependent on the expression status of E2F1 or Myc.

## Discussion

FTO is originally considered as a body mass and obesity associated gene, which mainly serves as a metabolic regulator [26–28]. The identification of FTO as a m^6^A demethylase has opened a new era for people to understand the function of FTO in different biological process [7], particularly in tumorigenesis. Increasing evidence revealed the oncogenic role of FTO in different cancer subtypes, for example, FTO triggers leukemic cell transformation and leukemogenesis via regulating expression of *ASB2* and *RARA* by reducing their mRNA transcripts’ m^6^A levels [31]; FTO is highly expressed in human melanoma and promotes melanoma tumorigenesis as well as anti-PD-1 resistance by regulating downstream targets including PD-1, CXCR4 and SOX10 [32]; in breast cancer, FTO accelerates breast cancer proliferation, colony formation and metastasis by regulating the transcript’s decay of a pro-apoptosis gene BNIP3 [33]; whereas in lung cancer, FTO decreases m^6^A level of ubiquitin-specific protease (USP7) therefore sustains the stability of USP7’s mRNA [34]. Although FTO’s functions or targets in various cancer subtypes are different, the oncogenic role of FTO can be attributed to two aspects, either promote oncogenes translation or trigger tumor suppressors’ transcripts decay, which is dependent on its demethylase activity.

In the present study, we showed that FTO was frequently overexpressed in cervical cancer tissues and positively correlated with tumorigenesis, functional FTO but not mutant FTO without demethylase activity could regulate cervical cancer cells’ proliferation and migration. Mechanistically, FTO directly interacted with E2F1 and Myc mRNAs and inhibition FTO dramatically impaired these two important oncogenes’ translation, thus suppressed cervical cancer cells’ proliferation and migration. Intriguingly, either overexpression of E2F1 or Myc could efficiently rescue the inhibition of cervical cancer cells’ proliferation or migration which induced by FTO’s deficiency, suggesting these two genes might regulate each other in cervical cancer cells, for instance, Myc was proved to induce transcription of E2F1 while inhibiting its translation via a microRNA polycistron [35]. Paradoxically, we also noticed some genes, especially p53 pathway associated genes were upregulated after knocking down of FTO, whether these genes were direct targets of FTO or FTO had other potential functions beyond m^6^A regulation required further investigation.

In line with previous studies, our study also identified FTO acted as an oncogenic factor which regulated cervical cancer cells’ proliferation and migration, thus, FTO becomes a potential target for cancer therapy which attracts intense research interest. For instance, two FTO inhibitors FB23 and FB23-2 were shown to inhibit FTO’s m^6^A demethylase activity, these compounds could mimic the FTO deficiency effect which dramatically suppressed proliferation as well as promoted differentiation/apoptosis in human acute myeloid leukemia (AML) both in and ex vivo [36]; another group reported the Food and Drug Administration-approved drug entacapone could directly bind and inhibit FTO in vitro, moreover, they confirmed that entacapone could decrease body weight and fasting blood glucose concentrations in obesity mice via FOXO1 [37], it will be quite interesting to investigate whether this available drug has anti-tumor effect in different cancer models.

Unlike many other developed targeted drugs which designed to bind cell surface receptors or transcription factors, the philosophy to target FTO for cancer therapy is to regulate oncogenes’ expression at translation level, the advantages are: (i) it can control many downstream targets simultaneously with potential synergistical effect; (ii) translational control is more efficient than transcriptional control when evaluated for protein outputs as it can avoid many compensation effects; (iii) one drug targets FTO might be useful for many more different cancers compare to classical targeted drugs. However, it is also apparent that the drugs targeted FTO may induce unexpected or unpredictable side effects when applied in vivo, as inhibition of m^6^A demethylation can affect many genes’ expression, the toxicity study for these drug candidates should cost extraordinary effort.

## Conclusions

Taken together, our study demonstrates that the m^6^A demethylase FTO plays an important role in regulating cervical cancer cell proliferation and migration, the oncogenic function of FTO is dependent on its demethylase activity. Our data further reveals that FTO can directly regulate the translation of two important oncogenic factors E2F1 and Myc, and overexpression of E2F1 or Myc sufficiently restores the proliferation and migration capacity in FTO deficient cells. We therefore provide direct evidence that targeting FTO signaling represents a promising strategy for cervical cancer therapy.

## Supplementary information

**Additional file 1: Table S1.** Differentially expressed genes in Hela cells with FTO knockdown.

## Data Availability

All datasets generated in this study was accessible upon reasonable requirement.
