# Correction to: The m^6^A eraser FTO facilitates proliferation and migration of human cervical cancer cells

**DOI:** 10.1186/s12935-020-01473-8

**Published:** 2020-08-30

**Authors:** Dongling Zou, Lei Dong, Chenying Li, Zhe Yin, Shuan Rao, Qi Zhou

**Affiliations:** 1 Chongqing Key Laboratory of Translational Research for Cancer Metastasis and Individualized Treatment, Chongqing University Cancer Hospital & Chongqing Cancer Institute & Chongqing Cancer Hospital, Chongqing, 400030 China; 2 Department of Systems Biology, Beckman Research Institute of City of Hope, Monrovia, CA USA; 3 Department of Hematology, School of Medicine, The First Affiliated Hospital of Zhejiang University, Hangzhou, 310003 China; 4 Department of Thoracic Surgery, Nanfang Hospital, Southern Medical University, Guangzhou, 510515 China

## Correction to: Cancer Cell Int (2019) 19:321 10.1186/s12935-019-1045-1

Following publication of the original article [1] the authors have notified us of a few errors in Figures 2, 3 and 6. The corrected Figs. 2, 3, and 6 are presented below.

**Fig. 2:**
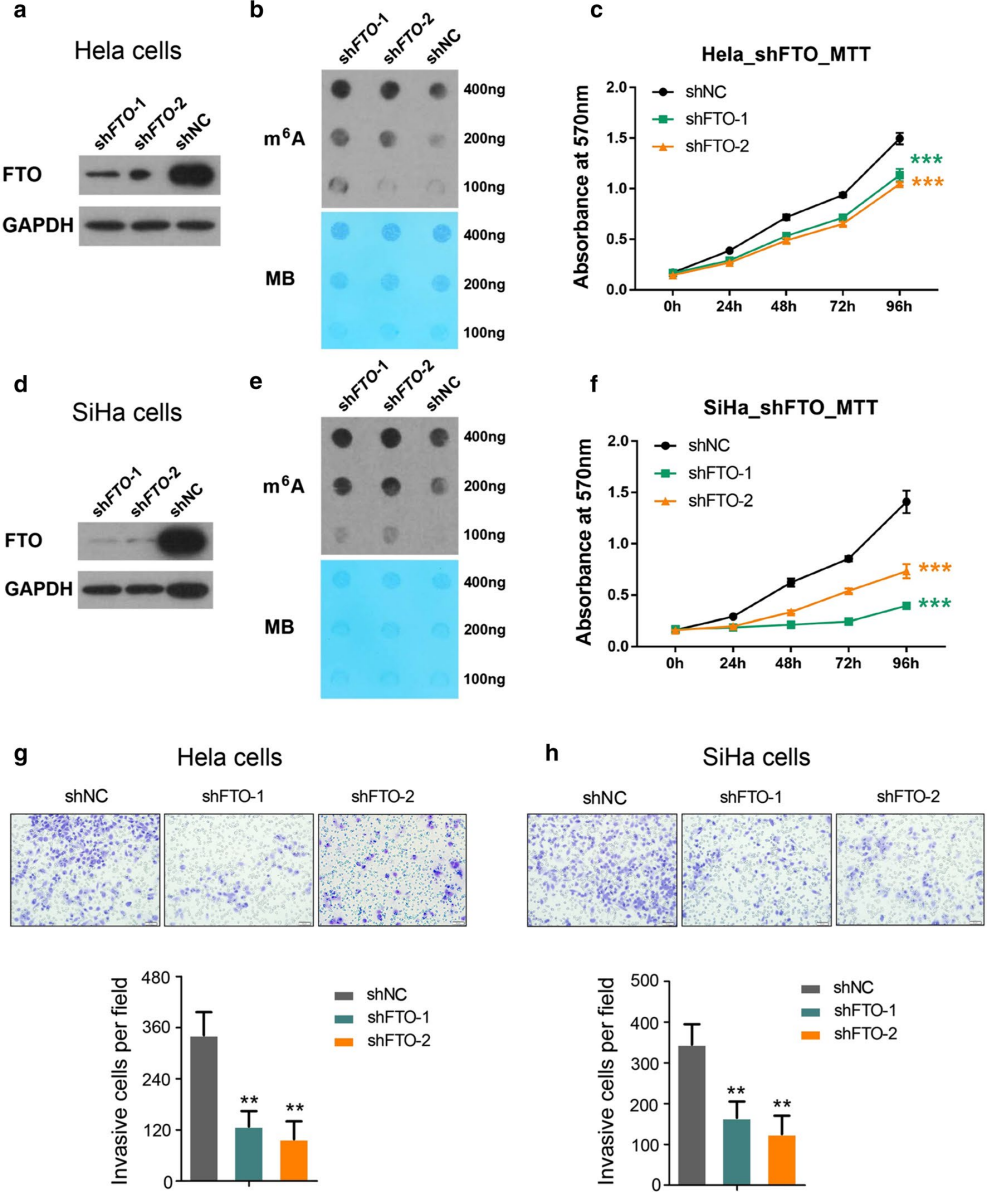
FTO regulates cervical cancer cells’ proliferation and migration. **a** Immunoblot analysis of FTO expression in control and knocking down Hela cells using two different shRNAs; **b** m^6^A dot blot assays of Hela cells with or without knocking down of FTO. MB, methylene blue staining (as loading control); **c** effects of knocking down FTO on Hela cells growth/proliferation. ***P < 0.001. (Student’s t test); **d** Western blot analysis of FTO expression in control and knocking down SiHa cells using same shRNAs as described in **a**; **e** m^6^A dot blot assays of SiHa cells with or without knocking down of FTO. MB, methylene blue staining (as loading control); **f** effects of knocking down FTO on SiHa cells growth/proliferation. ***P < 0.001. (Student’s t test); **g**, **h** analysis of cell migration capacity using competent or deficient Hela and SiHa cells

**Fig. 3:**
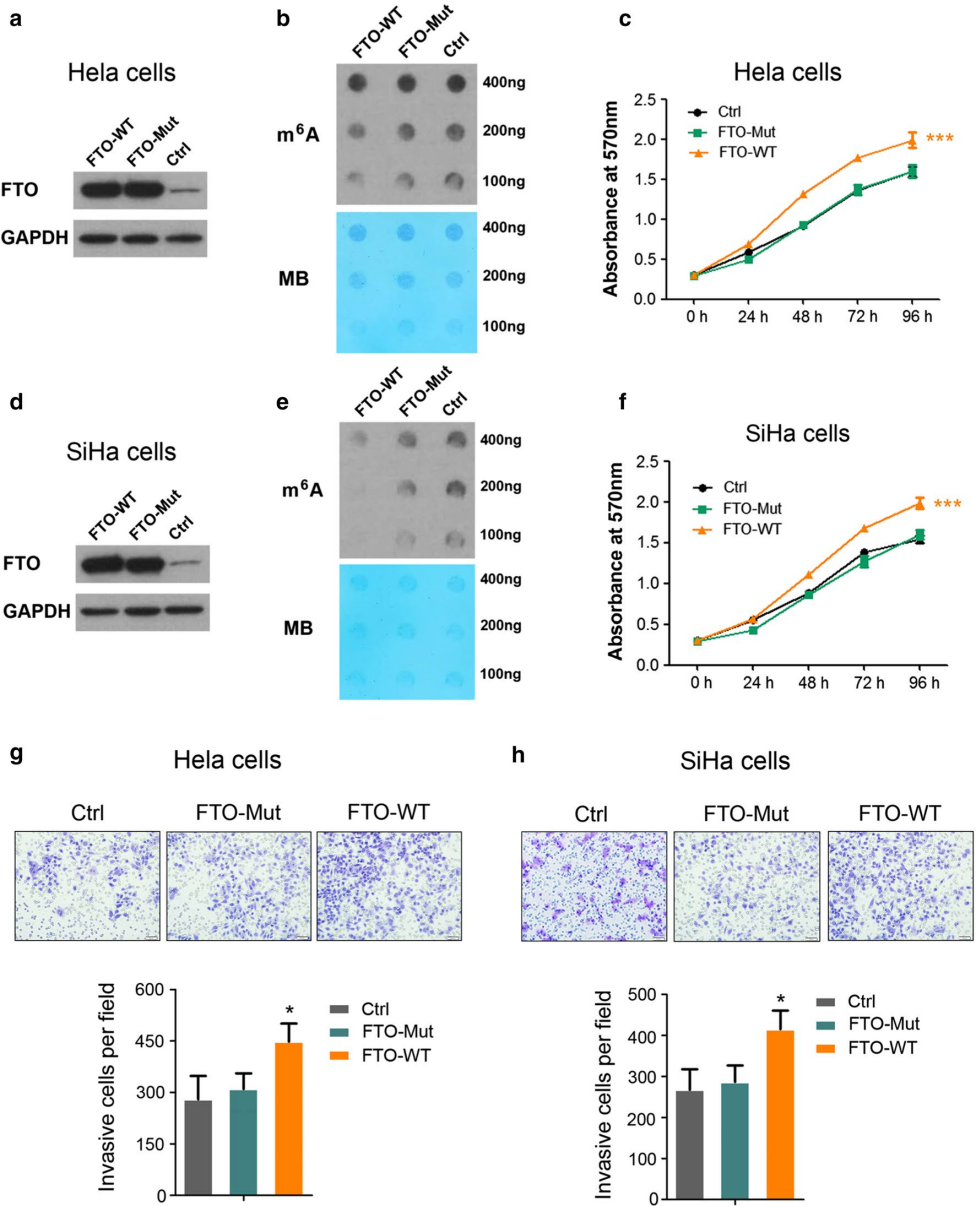
The m^6^A demethylase activity is required for FTO to play its oncogenic function. **a** Enforced FTO or FTO-mut expression in Hela cells. FTO-mut carries two point- mutations, H231A and D233A, which disrupt the enzymatic activity of FTO. GAPDH was used as a loading control; **b** m^6^A dot blot assays of Hela cells with enforced FTO or FTO-mut expression. MB, methylene blue staining (as loading control); **c** effects of FTO or mutant FTO overexpression on Hela cells’ growth/proliferation, ***P < 0.001. (Student’s t test); **d** enforced FTO or FTO-mut expression in SiHa cells. GAPDH was used as a loading control; **e** m^6^A dot blot assays of SiHa cells with enforced FTO or FTO-mut expression. MB, methylene blue staining (as loading control); **f** effects of FTO or mutant FTO overexpression on SiHa cells’ growth/proliferation, ***P < 0.001. (Student’s t test); **g**, **h** analysis of cell migration capacity in FTO or mutant FTO overexpressed Hela and SiHa cells

**Fig. 6:**
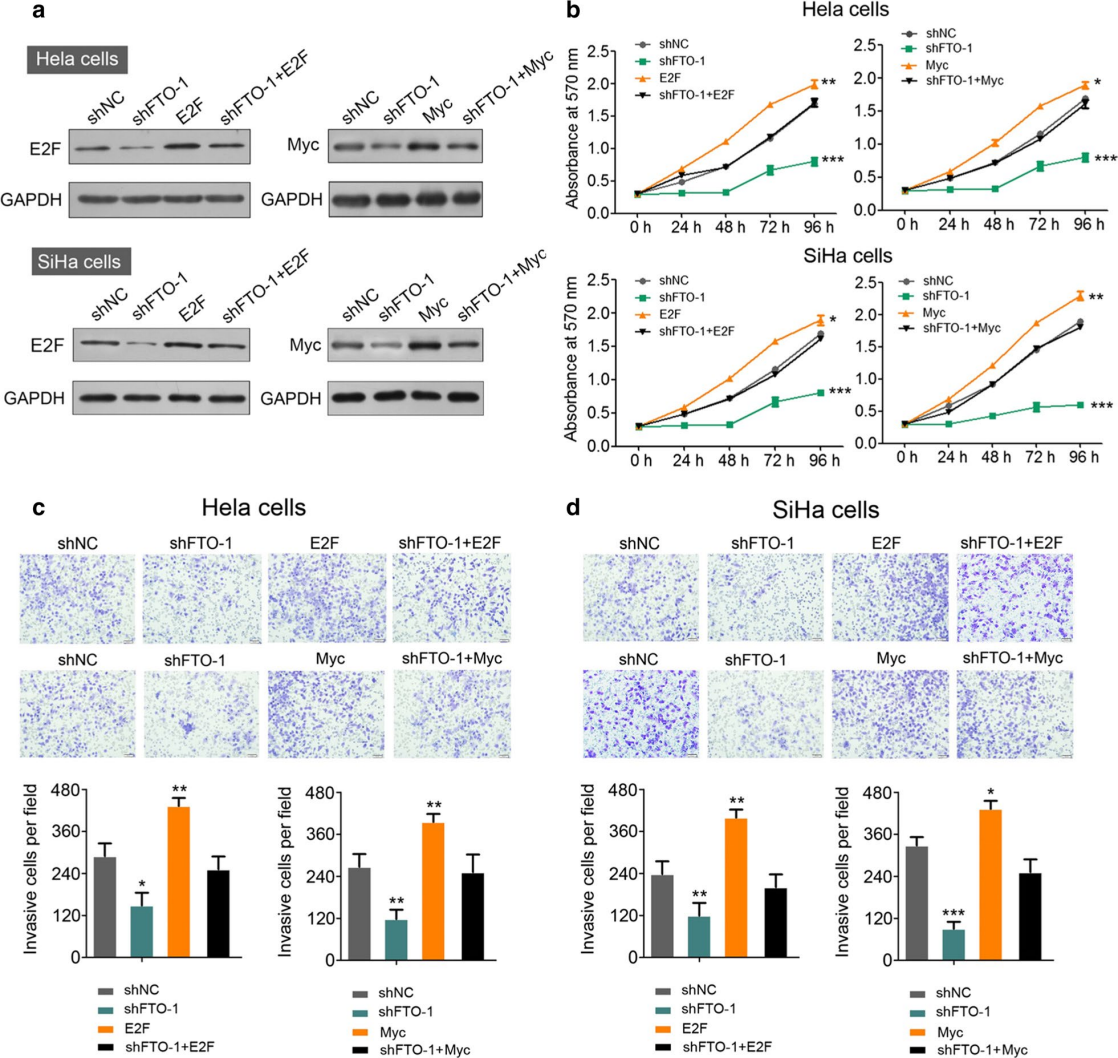
Overexpression of E2F1 or Myc compensates FTO knocking down effect. **a** The protein level determination of overexpressed E2F1 or Myc in FTO control and knockdown Hela and SiHa cells, **P < 0.01 (unpaired two-sided t test); **b** cell proliferation analysis of Hela (upper panel) and SiHa cells (lower panel) in different genetic background as described (**a**). *P < 0.05, **P < 0.01, ***P < 0.001 (Student’s t test). **c**, **d** The migration analysis of Hela cells (**c**) and SiHa cells (**d**) in different genetic background as described in **a**
